# Fostering continuous quality improvement in a European rare disease network: Where are we now?

**DOI:** 10.1186/s13023-026-04324-3

**Published:** 2026-04-29

**Authors:** Linde Margriet van der Kamp, Sara Román Galdrán, Willemijn F. E. Irvine, Daniel Rossi, Romee van Steekelenburg, ERNICA Representatives, René M. H. Wijnen, Erwin Ista, Olivia K. C. Spivack

**Affiliations:** 1 European Reference Network for Rare Inherited and Congenital Anomalies (ERNICA), Rotterdam, The Netherlands; 2 Department of Pediatric Surgery, Erasmus MC Sophia Children’s Hospital, Erasmus University Medical Center Rotterdam, Rotterdam, The Netherlands; 3 Dutch Institute for Clinical Auditing (DICA), Leiden, The Netherlands; 4 Department of Evidence Based Medicine and Methodology, Qualicura Healthcare Support Agency, Breda, Netherlands; 5 Department of Women’s and Children’s Health, Karolinska Institutet, Stockholm, Sweden; 6 Department of Neonatal and Pediatric Intensive Care, Division of Pediatric Intensive Care, Erasmus MC Sophia Children’s Hospital, Erasmus University Medical Center Rotterdam, Rotterdam, The Netherlands; 7 Department of Internal Medicine, Section of Nursing Science, Erasmus MC, Erasmus University Medical Center Rotterdam, Rotterdam, The Netherlands

**Keywords:** Cross-border collaboration, Rare diseases, Quality improvement, Clinical practice guidelines, Implementation science, European reference network, ERNICA

## Abstract

**Background:**

The European Reference Network for rare Inherited and Congenital Anomalies (ERNICA) aims to improve care for patients with rare and complex digestive and gastrointestinal diseases across Europe through cross-border collaboration. In a previous publication, we introduced the ERNICA quality cycle as a structured framework for the continuous improvement of care. This framework focuses on five steps: Describing the desired quality of care, promoting implementation, measuring care quality, evaluating clinical practice and performing research.

**Main body:**

Focusing on three selected ERNICA conditions (omphalocele, rectosigmoid Hirschsprung’s disease and esophageal atresia), this paper reports on progress made across all steps of the ERNICA quality cycle. Activities include the development of clinical guidelines and core quality indicator sets and efforts to standardise clinical definitions. To support successful guideline implementation, both a central implementation support team and a network of local implementation leads have been established. Quality measurement is facilitated through the European Pediatric Surgical Audit (EPSA), which enables real-world data collection, benchmarking and the evaluation of clinical practice. Steps have been taken to identify and fill knowledge gaps through high-quality research. Efforts include the development of core outcome sets, exploring the use of EPSA data for research purposes, and sharing lessons learnt from ERNICA-endorsed randomised controlled trials.

**Conclusion:**

The progress presented in this paper reflects ERNICA’s growing impact and sustained dedication to improving care and outcomes for patients with rare digestive and gastrointestinal diseases across Europe. This update aims to keep stakeholders informed, promote transparency, and facilitate continuous learning and collaboration. Building on lessons learnt, future efforts will involve optimising ongoing initiatives and scaling the ERNICA quality cycle to include additional diseases. Although these efforts are not without challenge, ERNICA remains committed to improving care quality across its connected centres and beyond.

## Background

**Fig. 1:**
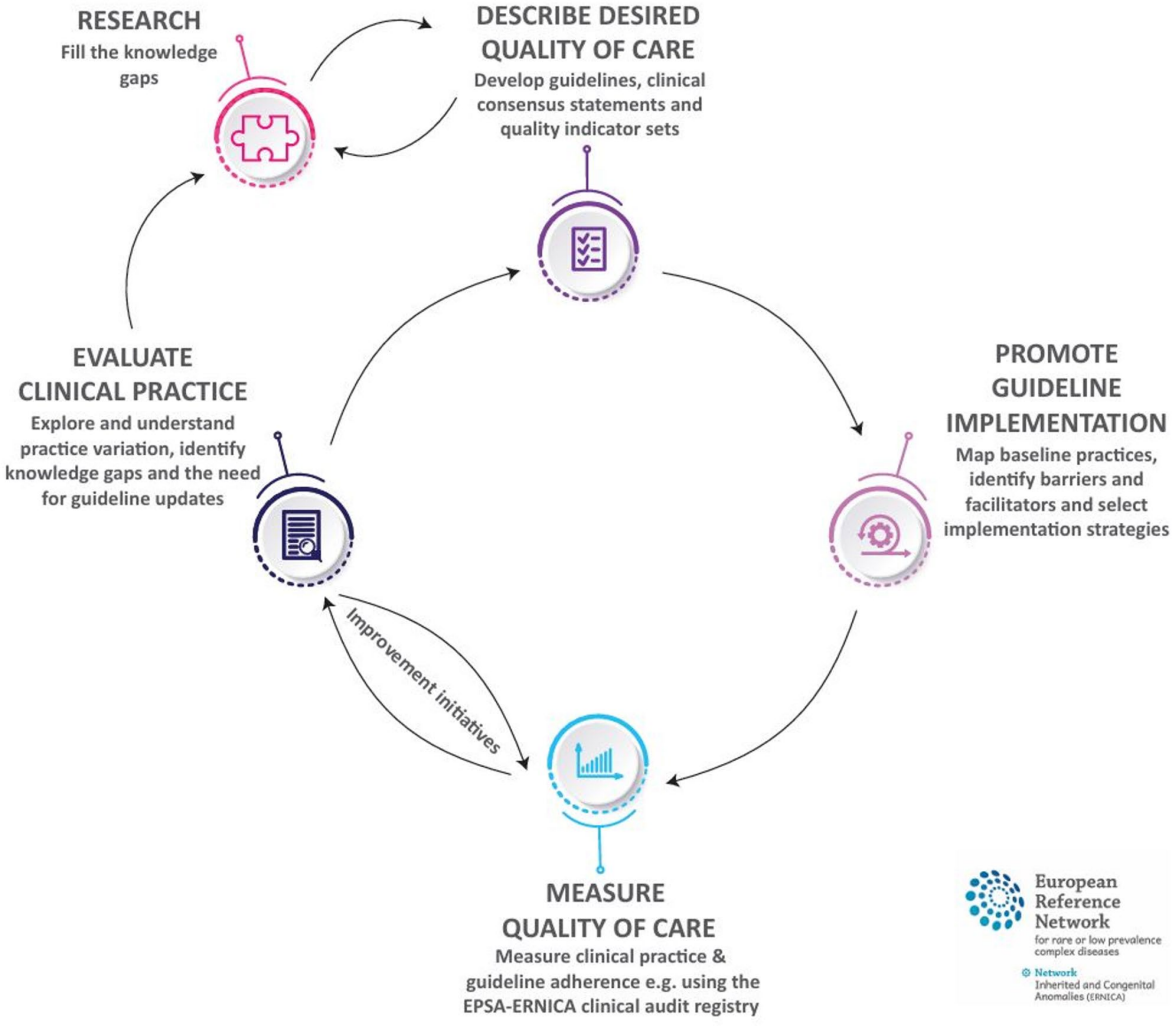
The ERNICA quality cycle

The European Reference Network for rare Inherited and Congenital Anomalies (ERNICA) was established by the European Commission in 2017 to improve care for patients with rare and complex digestive and gastrointestinal diseases in Europe [1]. By bringing together multidisciplinary experts from specialised centres, ERNICA facilitates knowledge sharing and cross-border collaboration. Since its conception, ERNICA has coordinated a wide range of activities to address the specific needs of its patient population and specialists. In our previous publication, *“Fostering continuous quality improvement in a European rare disease network”* [2], we introduced the ERNICA quality cycle as a structured approach to improving care for patients with rare and complex digestive and gastrointestinal diseases, and reflected on its application and evaluation. This five-step framework involves describing the desired quality of care (e.g., through guideline development), promoting guideline implementation, measuring care quality and evaluating clinical practice, ultimately informing the need for additional research, which may lead to new guidelines and revisions (see Fig. 1 – The ERNICA quality cycle). In this paper, we reflect on progress made at all steps of the cycle, focusing on three diseases for which ERNICA guidelines or consensus statements are available or soon to be published: omphalocele, rectosigmoid Hirschsprung’s disease and esophageal atresia. In doing so, we aim to document ERNICA’s growing impact and reaffirm our commitment to improving the quality of care for patients with rare digestive and gastrointestinal diseases in Europe. Tables 1, 2 and 3 have been included to guide the reader through the paper. These tables include a list of abbreviations used (Table 1), a key word list for specific terms (Table 2), complete with definitions or explanations, and a list of ongoing or published studies related to the activities described (Table 3).

**Table 1:** List of abbreviations used

Abbreviation	Full name
ERN	European Reference Network
ERNICA	The European Reference Network for rare Inherited and Congenital Anomalies
EPSA	European Pediatric Surgical Audit
GRADE	Grading of Recommendations Assessment, Development and Evaluation
REGARD	Real-World Evidence for Guidelines Addressing Rare Diseases
CheckUp	Checklist for the Reporting of Updated Guidelines
HAEC	Hirschsprung’s Associated Enterocolitis
EUPSA	European Pediatric Surgeons’ Association
AGREE-REX	Appraisal of Guidelines REsearch and Evaluation – Recommendations Excellence
CIST	Central Implementation Support Team
DICA	Dutch Institute for Clinical Auditing
PROs	Patient-Reported Outcomes
PROMs	Patient-Reported Outcome Measures
ePAG	European Patient Advocacy Group
OCELOT	Oesophageal atresia CorE outcomes LOng Term
RCT	Randomised-Controlled Trial
C4C	Conect4Children
ECET	European Consortium for Epilepsy Trials
MWPSC	Midwest Pediatric Surgery Research Consortium

**Table 2:** Key word list for ERNICA-specific terms

Key word	Definition/explanation
Structured observation form	“A matrix that experts use to rate their perceived effectiveness of an intervention on selected outcomes on a spectrum from harmful to beneficial using a Likert type scale” [3].
ERNICA guideline methodologist	ERNICA team member whose role is focused on preparing and steering the development of disease-specific clinical practice guidelines and other clinical decision support tools such as clinical consensus statements. The ERNICA guideline methodologist is responsible for coordinating the panel of experts involved in guideline development.
ERNICA implementation coordinator	ERNICA team member whose role is focused on coordinating efforts in pursuit of the successful implementation of ERNICA clinical guidelines/consensus statements, PROMs and the use of clinical auditing (EPSA registry) as a quality improvement tool. The ERNICA implementation coordinator is responsible for coordinating the ERNICA CIST and local implementation leads network.
The ERNICA Central Implementation Support Team (CIST)	A transversal, cross-disease working group within ERNICA dedicated to supporting the successful implementation of ERNICA guidelines, PROMs and the use of clinical auditing (EPSA registry) as a quality improvement tool.
The ERNICA local implementation leads network	A group of clinical representatives who have been appointed locally to serve as ‘implementation leads’ for their hospital. Local implementation leads serve as the local point of contact for the CIST and help in identifying local team members/stakeholders to be involved in specific implementation projects. They maintain a high-level overview of the ERNICA-led implementation initiatives relevant to their hospital.
Codman dashboard	A dashboard that is updated weekly, displaying information related to a hospital’s performance compared to other hospitals in an anonymised way. Performance is shown using funnel plots with 95% confidence intervals around the benchmark. This allows a hospital to compare their own outcomes to other European centers. Besides, it generates lists of patients meeting the criteria for specific quality indicators, for example, those who developed a specific complication. These lists allow participating professionals to analyse the disease course, treatment, and actions that led to desirable or unwanted events in a specific patient group. The exploratory dashboard allows a user to set filters, to take a closer look at outcomes of specifically selected patient groups [4].
EPSA annual feedback sessions	An annual, structured feedback session focused on a specific disease area, during which registry data are systematically analysed and benchmarked. Relevant outcomes, trends, and inter-institutional variation are reviewed and discussed in a multidisciplinary setting. The objective is to translate data-driven insights into concrete quality improvement goals, stimulate shared learning, and promote continuous improvement of care [4].
Patient journey	ERNICA patient journeys describe the key stages in the life of a person living with a rare inherited or congenital condition, from possible prenatal diagnosis through to adulthood. Co-developed by patient representatives and clinicians, they provide an overview of symptoms, diagnosis, treatment, and good clinical and support practices at each stage, using accessible language. Their purpose is to raise awareness of the patient experience, promote understanding and support effective communication between patients, families, and clinicians [5].
ERNICA Patient Advocacy Group (ePAG)	The ERNICA European Patient Advocacy Group (ePAG) is a collective of European patient organisations that are formally involved in the network and ensure that patient experiences and priorities are integrated into ERNICA’s deliverables and strategic decision-making. At the time of publication of this article, the ERNICA ePAG includes 15 patient organisations from across Europe.

**Table 3:** List of ongoing or published studies as described

ERNICA study	Current status
*The ERNICA quality cycle as a cyclical approach to quality improvement*	Published [2]
**Describing the desired quality of care**	
**Omphalocele**	
Development of a clinical guideline	
*ERNICA evidence-based guideline on omphalocele*	Accepted for publication [6]
*Use of EPSA data as a supplement to published evidence during the guideline development process for omphalocele (the REGARD method)*	Published [3]
Development of a core quality indicator set	
*Development of an internationally validated core quality indicator set through a systematic review and Delphi consensus study*	Systematic review and Delphi studies underway [Planned finalisation in 2026]
**Hirschsprung’s disease**	
Development and revision of a clinical guideline	
*ERNICA clinical guideline on the management of rectosigmoid Hirschsprung’s disease (original guideline)*	Published [7]
*Updated ERNICA guideline on the management of rectosigmoid Hirschsprung’s disease*	Accepted for publication [8]
Development of a core quality indicator set	
*Systematic review*	Published [9]
*Delphi consensus study*	Published [10]
Agreement on a standardised definition for Hirschsprung’s Associated Enterocolitis (HAEC)	
*A new expert opinion based clinical definition and severity grading of Hirschsprung’s Associated Enterocolitis*	Submitted for publication [11]
**Esophageal Atresia**	
Development and revision of clinical consensus statements	
*ERNICA consensus statements on the management of patients with esophageal atresia and tracheoesophageal fistula*	Published [12–14]
*Updated ERNICA consensus statements on the management of esophageal atresia and tracheoesophageal fistula*	Underway [Planned finalisation in 2026]
Development of a core quality indicator set	
*Systematic review*	Published [15]
*Delphi consensus study*	Published [16]
**Promoting guideline implementation**	
Implementation efforts during the guideline development process	
*ERNICA evidence-based guideline on omphalocele*	Accepted for publication [6]
*Updated ERNICA guideline on the management of rectosigmoid Hirschsprung’s disease*	Accepted for publication [8]
Addressing ERNICA’s implementation agenda	
*Omphalocele “baseline” clinical practices study*	Submitted for publication [17]
*Rectosigmoid Hirschsprung’s disease “baseline” clinical practices study*	Submitted for publication [18]
*Omphalocele focus group study assessing possible barriers and facilitators to clinical guideline implementation*	Underway [Planned finalisation in 2026]
*Esophageal Atresia “baseline” clinical practices EUPSA-ERNICA study*	Published [19]
Patient-friendly versions of guidelines and consensus statements	
*x 3 esophageal atresia patient-friendly versions of ERNICA consensus statements on the management of patients with esophageal atresia and tracheoesophageal fistula*	Published [20]
*Collaborative summary of existing care recommendations for respiratory complications in esophageal atresia*	Published [21]
**Measuring quality of care**	
The European Pediatric Surgical Audit (EPSA)	
*Study mapping the extent to which ERNICA guideline recommendations are suitable for measurement in a clinical audit*	Underway [Planned finalisation in 2026]
Challenges and points for consideration	
*How to assess, maintain and sustain data quality: an overview of completeness and accuracy of four years of registering the Dutch branch of the European Pediatric Surgical Audit*	Published as part of doctoral thesis [22]
*International data verification study*	Underway [Planned finalisation in 2026]
The use of Patient-Reported Outcome Measures (PROMs)	
*ERNICA-EUPSA study exploring healthcare professionals’ current use of PROMs for rare digestive and gastrointestinal surgical conditions and their preferences for the future*	Underway [Planned finalisation in 2026]
**Evaluating clinical practice**	
Use of the EPSA	
*Updated ERNICA consensus statements on the management of esophageal atresia and tracheoesophageal fistula*	Underway [Planned finalisation in 2026]
Identifying research gaps and priorities	
*Rectosigmoid Hirschsprung’s disease patient journey*	Published [5]
*Esophageal atresia patient journey*	Published [5]
*Validation/revision of the Esophageal atresia patient journey*	Underway [Planned finalisation in 2026]
*Rectosigmoid Hirschsprung’s disease “baseline” clinical practices study*	Submitted for publication [18]
*Esophageal Atresia “baseline” clinical practices EUPSA-ERNICA study*	Published [19]
*Study to explore top ten research priorities for esophageal atresia developed by the esophageal diseases working group - How do these research priorities compare to those of patients and parents?*	Planned
**Research**	
Challenges conducting research in the field of rare (pediatric) diseases	
*Study on the experiences and lessons learned from two ERNICA-endorsed RCTs*,* recognising the challenges and proposing possible solutions*	Published [23]
ERNICA efforts to mitigate identified challenges	
*A new expert opinion based clinical definition and severity grading of HAEC*	Submitted for publication [11]
*Study on the experiences and lessons learned from two ERNICA-endorsed RCTs*,* recognising the challenges and proposing possible solutions*	Published [23]
*International data verification study*	Underway [Planned finalisation in 2026]
*ERNICA involved in: Development of a core outcome set for esophageal atresia (OCELOT study)*	Published [24]

## Describing the desired quality of care

Describing the desired quality of care is a core step in the ERNICA quality cycle [2]. This step involves the development of clinical guidelines, clinical consensus statements and core quality indicator sets. While a core outcome set seeks to standardise data collection for research purposes [25], a core indicator set consists of indicators that measure the quality of care provided throughout the care pathway. Quality indicators can record information at structural, process and outcome level [2, 4].

### Omphalocele

#### Development of a clinical guideline

Progress has been made in describing the desired quality of care for omphalocele, through the development of a clinical guideline [6]. Historically, clinical guidance for this rare condition has been missing, which has resulted in a high level of clinical practice variation [6]. To develop this clinical guideline, an international, multidisciplinary guideline panel was established, involving clinical experts and patient representatives. The GRADE Evidence-to-Decision framework [26] guided the development process. To mitigate the lack of high-quality evidence for this rare condition, traditional guideline methodology was supplemented with an innovative approach (the Real-World Evidence for Guidelines Addressing Rare Diseases [REGARD] Method) [3] adapted from Pai et al. [27]. Uncorrected real-world data from the European Pediatric Surgical Audit (EPSA) were presented to the guideline panel, who individually completed structured observation forms designed to capture their interpretation of how specific interventions relate to named outcomes. These forms were reviewed by ERNICA’s guideline methodologist (W.F.E.I), who presented the findings during the evidence-to-decision phase of the guideline development process. Although not without limitations, this approach showed promise as a methodological supplement in guideline development for rare conditions with a lack of high-quality evidence [3].

#### Development of a core quality indicator set

Work is underway to develop an internationally validated core quality indicator set for omphalocele, through a systematic review and Delphi consensus study. This quality indicator set, alongside other sources such as ERNICA’s new clinical guideline, will help inform revision of the dataset for omphalocele in the EPSA. The resulting dataset aims to be both comprehensive and practical, enabling consistent data collection across centres for benchmarking, quality monitoring and research purposes, while also facilitating the assessment of guideline adherence.

### Rectosigmoid Hirschsprung’s disease

#### Development and revision of a clinical guideline

ERNICA’s guideline for rectosigmoid Hirschsprung’s disease has recently undergone monitoring and subsequent revision. In 2020, ERNICA published the first European clinical guideline for the management of this disease [7]. With ERNICA guidelines assessed for revision every five years [2], this disease-specific guideline was recently updated in 2025 and is awaiting publication [8]. This revision process involved priortising recommendations to update, conducting up-to-date literature searches to identify new contributing evidence and holding an international, multidisciplinary panel discussion meeting. The update process was guided by the GRADE evidence-to-decision framework [26] and Checklist for the Reporting of Updated Guidelines (CheckUp) framework [28], and involved a panel of both clinical experts and patient representatives.

#### Development of a core quality indicator set

Recommendations included in ERNICA’s original rectosigmoid Hirschsprung’s disease guideline were used to inform the development of a core quality indicator set. This involved a rigorous process including a systematic review [9] and Delphi procedure [10]. This quality indicator set, alongside the available core outcome set [29], the updated guideline and other relevant sources, will be used to inform revision of the dataset for Hirschsprung’s disease in the EPSA.

#### Agreement on a standardised definition for Hirschsprung’s Associated Enterocolitis (HAEC)

There can be large variability in how clinical terms are defined, which can pose challenges during both the guideline and quality indicator development processes [2]. Steps have been taken within ERNICA to agree on a standardised definition for HAEC, grounded in expert opinion and real-world clinical experience. A mixed-methods design combining an initial qualitative study and modified Delphi approach was employed to reach agreement on a standardised definition and severity grading system for HAEC, involving a European, multidisciplinary group of experts [11].

### Esophageal atresia

#### Development and revision of clinical consensus statements

In 2020 and 2021, ERNICA published a set of three consensus statements for the management of esophageal atresia and tracheoesophageal fistula [12–14]. Revision of the esophageal atresia consensus statements is planned for 2026.

#### Development of a core quality indicator set

The published esophageal atresia consensus statements were used to inform the establishment of a core quality indicator set, developed following a systematic review and Delphi study [15, 16]. This quality indicator set, alongside a core outcome set developed in parallel [24], was used to establish a revised dataset for esophageal atresia in the EPSA. This revised dataset became operational on January 1, 2024, and includes both short-term outcomes and structured follow-up at one and two years.

### Cross-disease efforts

#### Strengthening collaboration with other networks

Strengthening collaboration with other scientific and professional networks remains a key priority for ERNICA. This will help to leverage shared expertise and resources, prevent duplication of efforts and enhance outreach. Steps are being taken to align guideline development efforts with those of other groups, such as the European Pediatric Surgeons’ Association (EUPSA).

## Promoting guideline implementation

### Implementation efforts during the guideline development process

This step in the cycle focuses on promoting the successful implementation of ERNICA’s clinical guidelines in clinical practice [2]. A dedicated ERNICA implementation coordinator (O.K.C.S) has been appointed to drive this mission forward. Efforts have been made to consider implementation during the guideline development process itself. ERNICA’s implementation coordinator has recently been involved in the guideline development panel for the ERNICA omphalocele guideline and the revision panel for the ERNICA rectosigmoid Hirschsprung’s disease guideline. During the GRADE evidence-to-decision process, the ERNICA implementation coordinator helped guide or expand on considerations pertaining to implementability. Where relevant, discussion was guided by aspects included in the AGREE-REX tool [30], a checklist developed to increase guideline implementability. The use of evidence-to-decision frameworks in themselves has also been found to improve scores on all domains of the AGREE-REX tool [31].

### Setting up a structure for implementation

An implementation structure has been established within ERNICA. A central implementation support team (the ERNICA CIST), managed by ERNICA’s implementation coordinator, has been set up, and local implementation leads from 18 ERNICA centres have been appointed [2]. This group of local leads has been formally established as ERNICA’s *‘local implementation leads network’.* An implementation agenda, developed by the CIST, has been shared and discussed with members of the local leads network, and a meeting structure has been defined.

### Addressing ERNICA’s implementation agenda

Active steps have also been taken to address initial components of ERNICA’s implementation agenda. This has involved two disease-specific studies (for omphalocele and rectosigmoid Hirschsprung’s disease), both focused on obtaining “baseline overviews” of current care practices in Europe [17, 18]. In these studies, current practices were compared to those recommended in ERNICA guidelines. The results will help to guide the allocation of appropriate support for guideline implementation, and may also prompt action from the healthcare teams. Moreover, results can be compared to future guideline adherence analyses as a measurement of implementation success. A similar EUPSA-ERNICA baseline study has also recently been published evaluating current practices related to the management of esophageal atresia [19].

These studies will serve as a starting point for implementation. As indicated in our original publication, efforts will now be made to explore barriers and facilitators to the successful uptake of the guideline recommendations in clinical practice, enabling the selection of implementation strategies [2]. In the case of the omphalocele guideline, a qualitative focus group study has been set up for this purpose. The baseline studies provide some initial insight into the reasons behind practices that deviate from those recommended. Insights captured by the omphalocele baseline study can therefore be triangulated with those identified in this focus group context. ERNICA’s local implementation leads played a crucial role in identifying appropriate multidisciplinary colleagues for participation in this study.

### Patient-friendly versions of developed guidelines and consensus statements

To maximise the reach of ERNICA-developed guideline and consensus statements, patient-friendly versions are developed, together with patient and parent organisations. These are currently available for two of ERNICA’s esophageal atresia consensus statements [20]. Patient representatives have also been instrumental in the development of a multidisciplinary, cross-ERN summary of existing care recommendations for respiratory complications in esophageal atresia [21].

## Measuring quality of care

### The European Pediatric Surgical Audit (EPSA) as a measurement tool

The EPSA is a clinical audit hosted by the non-profit Dutch Institute for Clinical Auditing (DICA) [4, 32]. It is active in collecting prospective data for multiple ERNICA diseases, including omphalocele, rectosigmoid Hirschsprung’s disease and esophageal atresia. Recent efforts have aimed to establish the EPSA as a continuous feedback mechanism for the measurement of guideline adherence. The EPSA team is integrating relevant recommendation-specific variables into the EPSA disease-specific datasets, to facilitate ongoing monitoring. A study is currently underway to map the extent to which ERNICA guideline recommendations are suitable for measurement in a clinical audit. We are also exploring ways in which we can understand the reasons behind non-adherence.

### Challenges and points for consideration

As previously acknowledged, use of the EPSA for quality improvement and monitoring purposes depends on the consistent and systematic registration of data by participating centres [2]. Initiatives are underway to improve the registration of specific variables, such as prenatal data, which are often difficult for pediatric surgeons to retrieve. Locally coordinated strategies, such as the use of structured summaries in electronic patient records, may support more complete and accurate data entry. In the future, local implementation leads may help to encourage the adoption of these summaries in clinical practice. Moreover, to ensure the quality and utility of the data collected in the EPSA, data verification is important to assess both data accuracy and completeness. So far, a data verification study focused on Dutch data collected between 2018 and 2021 has been performed [22]. As data is now registered internationally, a broader international data verification project is in progress to ensure data quality across all participating centres. In the future, the local implementation leads may play a part in fostering active audit participation and ensuring high-quality data registration.

### The use of Patient-Reported Outcome Measures (PROMs)

Patient-Reported Outcomes (PROs) are not currently captured by the EPSA [2]. Yet, Patient-Reported Outcome Measures (PROMs) can and have been used for quality improvement purposes in other settings [33]. A joint ERNICA-EUPSA study is underway to explore healthcare professionals’ current use of PROMs for rare digestive and gastrointestinal surgical conditions and their preferences for the future. Insights from this study will help both ERNICA and EUPSA to shape meaningful PROM initiatives moving forward, which may involve incorporating PROMs into the EPSA. Reflecting the shift in focus from mortality to morbidity, long-term follow-up data is already being measured for esophageal atresia and is planned to be measured for the diseases in due course.

### Using EPSA to drive care improvements

Using the EPSA, ERNICA intends to drive improvements in care quality, through reflection, discussion and knowledge exchange. In this regard, ERNICA benefits from DICA’s experience and expertise. ERNICA continues to make use of the Codman dashboard developed by DICA, which visualises between-hospital variation using funnel plots that display each hospital’s outcomes relative to anonymised centres. These plots include 95% confidence intervals around a benchmark, enabling healthcare professionals to interpret variation within a meaningful statistical context [34]. To engage clinicians and promote data-driven improvement, EPSA annual feedback sessions also continue to be organised, supporting learning and quality improvement, and demonstrating the value of audit participation. Centre practices and outcomes, benchmarked against other centres, are presented and discussion is encouraged to reflect on the variation observed and identify opportunities for improvement. In 2024, a feedback session was held on the topic of omphalocele, followed by one national and one international session in 2025 dedicated to Hirschsprung’s disease. These sessions highlighted the importance of complete data registration and inspired participants to further explore the reasons behind unexpected outcomes.

## Evaluating clinical practice

Evaluating clinical practice is a critical step in the ERNICA quality cycle [2]. Re-auditing centre practices allows us not only to assess the success of our implementation efforts and refine our implementation strategies, but also to flag the need for future research, new clinical guidelines and guideline updates.

### Use of the EPSA

A key achievement has been the establishment of EPSA’s exploratory Codman dashboard [32], which can provide insight into centre practices and associated outcomes. This dashboard allows users to select specific outcomes and apply patient-level or practice-based filters for detailed subgroup analyses. This flexibility is particularly valuable in our rare disease context, where case-mix correction is often not feasible due to small patient numbers. However, it also presents a challenge; applying very specific filters may result in subgroups too small to allow for meaningful comparison.

Future plans include, as previously mentioned, the revision of the esophageal atresia consensus statements, scheduled for 2026. It is the intention to use existing EPSA data to help identify areas of practice variation and prioritise areas for guideline revision. Insights into practice variation may also help to identify areas for future research and implementation efforts.

### Identifying research gaps and priorities

Research gaps and priorities are identified through various means within ERNICA. Whilst we work to establish the EPSA as a tool for guideline monitoring and evaluation, insights behind deviating practices, as captured by the baseline studies, can be used to identify areas for future research. During the rigorous guideline development and revision processes, knowledge gaps are also evident. Patient journeys are another valuable instrument to identify and prioritise areas for patient-centred research [2]. These have been developed for rectosigmoid Hirschsprung’s disease and esophageal atresia, with the latter currently under review [5]. Further, research prioritisation efforts are performed within the ERNICA disease-specific and transversal working groups. The ERNICA working group for esophageal diseases has systematically reviewed the top ten research priorities for their disease area. A study has been planned to explore how these research priorities compare to those of patients and parents. Patient representatives who are part of the ERNICA Patient Advocacy Group (ePAG) will have an important role in identifying patients and parents who were not involved in the working group prioritisation exercise for participation in this study. Other disease-specific working groups will be encouraged to conduct similar exercises.

## Conducting research

Producing high-quality evidence to fill identified knowledge gaps is instrumental in prompting a re-assessment of the desired level of care and restarting the iterative process [2]. Whereas the previous ‘evaluation’ step covers identifying areas for possible research, this step focuses on conducting it.

### Challenges conducting research in the field of rare (pediatric) diseases

ERNICA has increased efforts to facilitate research initiatives across Europe and foster multi-centre cross-border collaboration. However, the number of studies conducted in our target population remains low. This may be attributed to the fact that rare disease research presents a series of unique challenges, especially when the pediatric population is involved [2]. These include low case numbers, ethical issues, a complex regulatory and legal landscape, obstacles in data sharing, lack of funding, and difficulties obtaining parental consent [2, 23].

Moreover, while being considered one of the most rigorous ways to demonstrate treatment efficacy [35], the difficulties associated with conducting rare disease research are particularly evident in randomised controlled trials (RCTs). A recent ERNICA publication reflects on the experiences and lessons learned from two ERNICA-endorsed RCTs, recognising some of the key challenges [23].

### ERNICA efforts to mitigate identified challenges

To overcome difficulties concerning heterogeneous outcome reporting [2, 25], ERNICA has been involved in the development of a core outcome set for esophageal atresia (OCELOT study) [24]. Consensus studies like the aforementioned HAEC-definition study [11] also help to promote consistent measurement. Moreover, the ERNICA working group on quality of life is developing a decision tree support tool to help guide clinicians and researchers on how to develop and select PROMs appropriately for research purposes.

The aforementioned publication reflecting on the experiences and lessons learned from two ERNICA-endorsed RCTs has also proposed a list of possible solutions to some of the key challenges identified [23]. The paper stresses the importance of international collaboration, which can be facilitated by networks like ERNICA, in alleviating the obstacles encountered in multi-centre RCTs. Besides having a central role in fostering international collaboration, ERNICA has the potential to further help investigators navigate the complexities of rare disease research, both with RCTs and other study designs. To fulfill ERNICA’s potential, we are exploring the possibility of establishing a research facilitation infrastructure, which can support researchers in the design and conduct of clinical studies. Inspiration has been drawn from existing research facilitation networks and initiatives, such as Conect4Children (C4C) [36], the European Consortium for Epilepsy Trials (ECET) [37], and the Midwest Pediatric Surgery Research Consortium (MWPSC) [38]. Instead of duplicating work, we intend to connect and collaborate when and where possible. By cooperating and learning from others’ experiences, we hope to build a one-stop shop for the convenience of investigators involved in ERNICA. In parallel, we are devoting efforts to being able to use real world EPSA data for research purposes. The aforementioned international data verification project will help to guide this process, providing insight into levels of data quality and completeness.

## Future perspectives for the ERNICA quality cycle

The ERNICA quality cycle is intended as a scalable framework. Building on the experiences gained in this disease-specific pilot, future efforts may focus on extending and adapting the cycle to additional ERNICA disease entities, while continuing to refine and evaluate its impact [2]. With this pilot being a novel approach to quality improvement within our context, ongoing process evaluation will be critical moving forward [2]. Structured evaluation will help to formally capture the factors underpinning success, as well as the key challenges faced by those involved. Such an evaluation will help to shape future directions and initiatives. Promoting sustainability of the approach, and fostering inclusivity remain key priorities [2]. With all ERNs working towards the shared goal of improving care for rare and complex diseases, there may be scope for ongoing cross-ERN collaboration. ERNICA remains open to such collaborations and is receptive to new ideas, suggestions, and the use of innovative technologies to support us on this mission.

## Conclusion

This paper provides an overview of progress made for three ERNICA diseases for which guidelines or consensus statements are available or soon to be published, related to each step of the ERNICA quality cycle. Whilst there are ongoing challenges to tackle, some disease-specific and some generic, significant progress has been made and we remain committed to advancing the quality of care for our patient group. By sharing these developments transparently, we aim to keep stakeholders informed, inspire others and encourage ongoing collaboration. Future efforts will focus on optimising and evaluating the ERNICA cycle, possibly extending its scope to additional ERNICA diseases.

## Data Availability

Data sharing not applicable to this article as no datasets were generated or analysed during the current study.
